# Assessment of Eating Habits and Physical Activity among Spanish Adolescents. The "Cooking and Active Leisure" TAS Program

**DOI:** 10.1371/journal.pone.0159962

**Published:** 2016-07-27

**Authors:** Elena Roura, Raimon Milà-Villarroel, Sara Lucía Pareja, Alba Adot Caballero

**Affiliations:** 1 Alicia Foundation, Barcelona, Spain; 2 Research group on methodology, methods and models of health and social outcomes; Universitat de Vic-Universitat Central de Catalunya (UVic-UCC), Vic, Spain; McMaster University, CANADA

## Abstract

Worldwide obesity has more than doubled in the last forty years. Even more worrying is the fact that the number of overweight and obese children and adolescents has considerably increased. Socioeconomic development, as well as educational, agricultural and marketing policies have significantly changed dietary and physical activity habits among the youngest, who are thus susceptible to develop chronic and disabling diseases such as diabetes, some cancers and cardiovascular disorders. Adolescence is a critical age, in which the adoption of healthy habits may have dramatic effects on the health state in adulthood. For this reason, prompt interventions are urgently required to prevent the onset of obesity in this time of life. In this regard, the CAL-TAS program from Alicia Foundation was born to combat obesity and promote healthy lifestyles in Spanish adolescents. A total of 2519 students, aged 13–14 years, from 79 schools distributed all over the 17 autonomous communities in Spain were asked to report through the CAL-TAS platform their food intake and physical activity over one week. The body mass index, the consumption of food and beverages, the intake of macronutrients and micronutrients, and the values obtained from the PAQ-A questionnaire, which evaluated physical activity, were analyzed. Twenty percent of the participants were overweight or obese. In general, adolescents did not or poorly respected the recommendations provided by the Spanish Society of Community Nutrition. For example, in more than half of the subjects, the ingestion of fruits and beverages was less than recommended, whereas the consumption of meat, baked goods and fried foods was excessive. Moreover, adolescents with higher body mass index also presented worse eating habits and more inactivity. In conclusion, Spanish adolescents present low adherence to recommendations provided by the Spanish Society of Community Nutrition (SENC) and by the World Health Organization. In order to prevent obesity-related disorders, effective educational programmes have to be designed. Indeed, adolescents and their families should be aware that the early adoption of healthy dietary habits and of a correct physical activity may strongly improve their future quality of life.

## Background

The World Health Organization (WHO) has classified childhood obesity as “one of the most serious public health challenges of the 21^st^ century”, having been associated with a variety of physical, social and psychological consequences. A high body mass index (BMI) is a major risk factor for cardiovascular disorders [1], type 2 diabetes [2], and pulmonary [3], hepatic [4], renal [5] and musculoskeletal [6] complications as well as certain cancers [7,8]. Moreover, excess weight in children is associated with reduced quality of life [9,10] and increased risk of negative emotional states [11,12], undesirable stereotyping [13], bullying and social isolation [14].

Childhood obesity has dramatically increased over the past decade. Globally, about 42 million children under age 5 are overweight (OW) or obese (OB), a 60% increase since 1990 [9]. In addition, 31 million of overweight or obese children are living in developing countries. Recent reviews show that Spain had the highest overweight and obesity rates- around 32%- in 4-year old preschoolers [15] and was among the top four countries in Europe (along with Cyprus, Greece and England) with the highest obesity rates in youth aged 10 to 18 [16].

A number of factors contribute to the epidemic of overweight and obesity in children and adolescents, which encompass a combination of genetic, metabolic, behavioral, environmental, socio-cultural, and socioeconomic components. In general, increased body weight may be a multifactorial process, although incorrect diet and/or insufficient physical activity (PA) can be considered two of the main modulatory factors [17]. Thus, diet modification and the promotion of PA constitute critical components of all strategies aimed to combat childhood overweight and obesity [17–19].

Evidence-based guidelines to combat childhood obesity recommend breast-feeding, fruit and vegetable intake, control of portion sizes and of sweetened drink consumption, PA and reduction of television viewing [15,18,19].

School-based interventions are a major channel for many childhood obesity prevention programmes. Transition to secondary school may involve new habits for adolescents, as they often may exercise more autonomy over their own food choice and have increased opportunities to access to unhealthy food, thus limiting parental influence on their dietary behavior [20]. Children spend many hours at school, which serves as a key entity through which crucial behavior changes aimed to reduce childhood obesity can be addressed [19,21,22].

Despite the large number of studies already published on the prevention of childhood obesity, recent reviews show that research gaps exist in the definition of effective interventions for adolescents [21–24]. Adolescents represent a unique risk group due to their increased nutritional needs, and their tendency to adopt inadequate nutrition and physical activity habits [24,25]. Moreover, specific strategies are needed for this age group, since habits (healthy or not) established during adolescence are likely to persist into adulthood [18].

Unidirectional messages [26] or predesigned interventional strategies promoting changes in adolescents' eating behavior and PA seem to have limited effectiveness, or a short-term impact. Recently, some studies have employed alternative approaches that involve the target adolescent population in the improvement of their own eating habits. This type of participative interventions in adolescents seems to be more effective and results in better outcomes than strategies whose activities and messages are previously and uniquely elaborated by the research team [18,27,28]. In line with this innovative approach, after having reviewed methodologies utilized in various school-based nutrition, educational and PA programs and taking into consideration WHO recommendations, the Program *Cooking and Active Leisure (CAL)- Tú y Alicia por la Salud (You and Alicia for Health*, *TAS)*, hereafter referred to as CAL-TAS) was developed [29]. The aim of the project was to analyze the eating and PA habits adopted by Spanish adolescents.

In this regard, this paper wants to provide an updated picture of the food consumption habits and the PA engagement in young adolescents (13–14 years old) in Spain, in order to design, implement and evaluate a comprehensive school-based intervention program.

## Methods

### Sample size and participants

The selection of the study sample was conducted at the national level, enrolling 79 schools (103 classes) belonging to the 17 Autonomous Communities of Spain, for a total of 2516 students in the third level of secondary school, aged 13–14 years.

**Fig 1:**
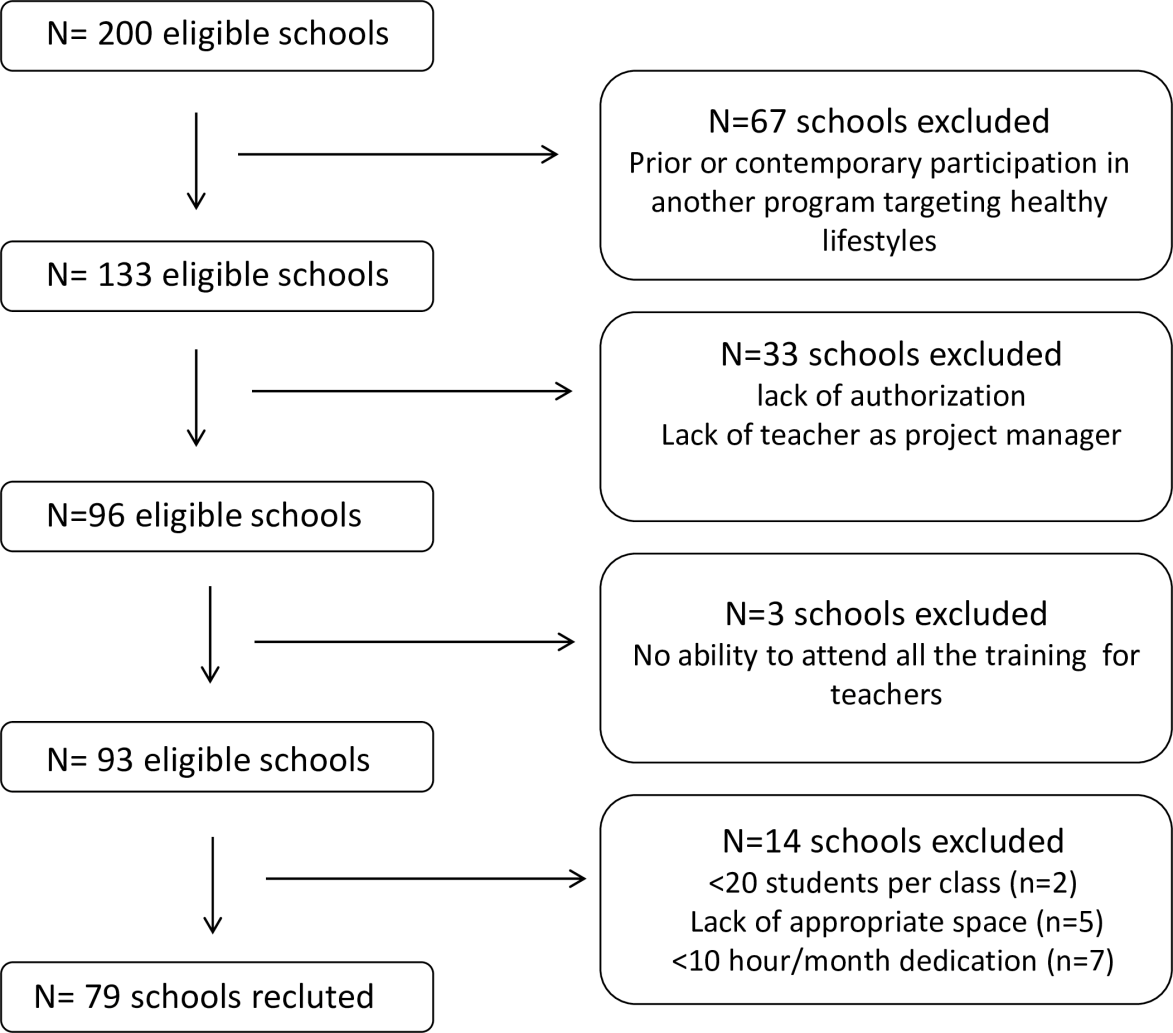
Flow Chart of the selection process performed to identify the schools admitted to participate to CAL-TAS programme. Exclusion criteria are indicated.

School recruitment was conducted in a randomized manner. Invitations to participate to the CAL-TAS study were sent to all schools nationally via the governmental agencies responsible for education in each of the Autonomous Communities. A total of 200 schools were enrolled, but only 79 were selected, according to the following criteria: a) no prior or contemporary participation in another promotion and/or education program targeting healthy lifestyles; b) presentation of an authorization for school participation and identification of a teacher designated to act as project manager; c) ability to attend all the training workshops for teachers, with the permission granted by the competent authority in each Autonomous Community; d) a minimum of 20 students per class; e) adequate space to conduct culinary and PA workshops; f) dedication of a minimum of 10 hours per month to the CAL-TAS program (Fig 1).

Neither socioeconomical status, nor geographical provenience (rural vs urban) have been considered in the selection process.

Of the 2516 subjects participating to the study, 299 were excluded due to incorrect completion of data registers, leaving a final study sample of 2217 participants.

### Data collection

A baseline study was conducted to evaluated students’ eating habits and PA. Each of the teachers from the participating schools explained to their students how to complete the food and PA registers. Students recorded their daily food and beverage intake over the course of one week using a standard 7-day dietary register[30]. Then, under teacher guidance and utilizing the supporting graphic materials provided, participants introduced on the CAL-TAS program's web platform the number of servings consumed. For each food recorded, a brief description of the preparation method and of ingredients, in the case of a recipe, was solicited. To quantify consumed foods and beverages, participants were asked to use household or standard consumption measures. To facilitate this task and to avoid bias in food recording, each participating group received several graphic materials describing household measures and/or standard consumption units in detail [29]. Students having access to cell phones with photographic capacity were also asked to upload photos of each consumed meal, so that experts from Alicia Foundation could quantify food and beverage rations.

Simultaneously, participants also completed the on-line PA register on the CAL-TAS program's web platform. Students were asked to answer the validated Physical Activity Questionnaire for Adolescents (PAQ-A questionnaire) that consists of 9 questions that evaluate distinct aspects of PA performed via a 5 point Likert scale [31]. The questionnaire was given to students during a class session and took approximately 10–15 minutes to be completed. Question 9 ask student to report if he was ill or whether some circumstances prevented him/her from realizing PA over the week. The final score was calculated as the arithmetic mean of scores recorded for the first 8 questions.

### Data analysis

All the following variables were analyzed: *Socio-demographic variables* included sex, age, academic year. The v*ariables referring to body mass index (BMI)* included: BMI value and percentage of participants belonging to one of the four following BMI categories: underweight (UW, BMI below percentile 5), normal weight (NW, BMI between percentile 5 and percentile 85), overweight (OW, BMI between percentile 85 to 95) and obese (OB, BMI above percentile 95). The v*ariables referring to PA included*: *participants were classified into one of these following categories*, *according to their PAQ-A score*:: very low (PAQ-A score <1), low (PAQ-A score >1 and <2), correct (PAQ-A score ≥2 and< 4) and high (PAQ-A score ≥4) PA levels; The variables referring to f*ood and drink consumption were*: number of servings consumed for each food (fruits, vegetables, legumes, dairy products, meat, white fish, blue fish, eggs, fried foods, baked goods), and drink group (soft drinks, alcohol), number of days without breakfast, calories consumed, macronutrients (grams of carbohydrates, proteins, lipids, and sugars) and micronutrients (calcium, iron, vitamin B6, vitamin B12, vitamin A, vitamin C, and vitamin D) consumed. For each food and beverage group, the percentage of participants belonging to one of the three categories 1) consumption less than recommended, 2) as recommended or 3) more that recommended has been provided, according to the dietary guidelines from the Spanish Society of Community Nutrition. Of note, recommendations on dietary consumption are the same for both females and males [32].

### Statistical analysis

The quantitative variables (food consumption variables, percentage of subjects according to dietary recommendations levels, diet quality indexes, PAQ-score) are described with measures of central tendency and dispersion such as the mean, standard deviation. In the case of non-normal distribution, the median and interquartile range was applied. Assessment of normality for these variables employed the Kolmogorov-Smirnov and Shapiro-Wilks tests, along with the QQ-Plot distribution graphics. For qualitative variables (socidemographic variables, variables associated with meals, knowledge and attitude variables, and score physical activity), absolute frequencies and percentages were utilized as descriptive means. To evaluate the differences between males and females according to the number of consumed food servings it has been used the comparison of independent means by t-test. To evaluate if there were significant differences in the macronutrients and micronutrients intake between both sexes, it has been used a simple regression model for each nutrient adjusted to the caloric intake and gender. To assess whether there were significant differences between nutrient consumption and food servings it was used the analysis of variance (ANOVA) with the SIDAK post-hoc tests. A 5% level of significance was considered for all performed analyses. Data analysis were carried out using SPSS v.18 and/or the open source R software. The mean comparisons and the analysis of variance has been carried out by SPSS v.18, and the lineal regression analysis by R software.

### Ethical approval of study and informed consent

This study has been approved by the ethics committee at University of Vic, Vic, Spain.

This study did not involve the collection of any primary data or access to any personal information related to the program’s participants or staff and thus was exempt from the requirements regarding informed consent.

## Results

### Study participants

Of the total number of subjects who correctly submitted the registers, 56% were females (n = 1241, age: 14.3 ± 0.88 years) and 44% were males (n = 973, age: 14 ± 0.61 years). The mean weight of the participants was 57.1 ± 10.5 kg (55.6 ± 10.20 kg for females and 59.71 ± 10.79 kg for males, p<0.05), and the mean height was 163 ± 13 cm (164.94 ± 8.85 cm for females and 1.66.33 ± 9.26 cm for males, p<0.05).

**Fig 2:**
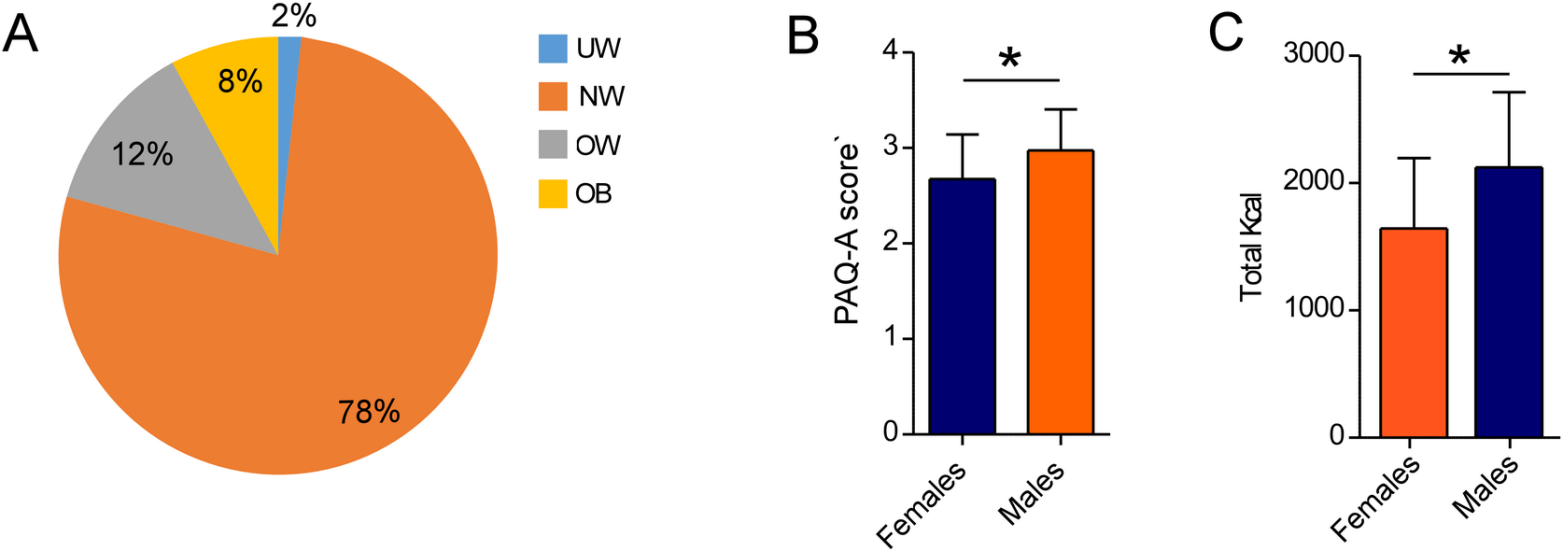
Overall description of participants. (A) Percentage of UW, NW, OW and OB adolescents participating to the study. (B) PAQ-A scores referring to the physical activity performed over one week, in females and males. Data are expressed as mean ± SD. (C) Amounts of calories ingested by females and males over one week. UW: underweight; NW: normal weight; OW: overweight; OB: obese. Data are expressed as mean ± SD, p = 0.0001.

The overall BMI was 20.8 ± 3.3 Kg/m^2^ and a statistically significant difference was observed between females and males (20.41 ± .24 and 21.55 ± 3.32, respectively, p<0.05). Of note, although most of the participants presented normal weight, one out of five was overweight or obese and one out of fifty was underweight (**Fig 2A**).

### Assessment of physical activity and food consumption habits

Spanish adolescents were asked to register their PA (expressed as PAQ-A score) and food and beverage intake over one week (expressed as number of servings and amount of consumed macronutrients and micronutrients). The overall PAQ-A score was statistically different in females and males, with the latter practicing more PA than their female peers (2.97 ± 0.74 and 2.68 ± 0.81, respectively, p<0.05) (**Fig 2B**).

On the other hand, the analysis of the energy consumption and eating habits of the participants reveals that higher amounts of ingested total calories were reported for males (2124 ± 590 Kcal/day vs 1644 ± 552 Kca/day, p<0.05), although in both groups, calorie intake was close to the reference values [33] (**Fig 2C**). In contrast, the number of servings for each food or beverage category was comparable between genders, even though males consumed more fried foods than females (3.53 ± 3.04 vs 3.32 ± 2.80, p = 0.09) (**Table 1**).

**Table 1:** Number of beverage and food servings ingested over one week and consumption adequacy according SENC recommendations.

	Females		Males			% adequacy to SENC recommendations		
	Mean	SD	Mean	SD	p-value	Below	Correct	Higher
Fruits	6.30	2.35	6.44	2.31	0.56	98,10	1,90	------
Vegetables	3.42	2.67	3.48	2.79	0.62	99,40	0,60	------
Legumes	1.60	1.60	1.59	1.62	0.88	54,90	41,20	3,90
Dairy products	9.46	3.92	9.74	2.68	0.26	78,30	20,90	0,70
Meat	6.50	3.38	6.70	3.67	0.21	9,40	22,90	67,70
White fish	2.09	1.68	2.03	1.85	0.47	67,90	23,20	8,90
Blue fish	0.96	1.15	0.91	1.26	0.26	44,90	46,20	8,90
Eggs	2.16	1.88	2.26	2.04	0.19	64,50	26,30	9,20
Fried foods	3.32	2.80	3.53	3.04	0.09	------	61,40	38,60
Soft drinks	5.09	2.14	5.30	2.42	0.36	------	47,30	52,70
Baked goods	5.09	2.15	5.23	2.32	0.54	------	47,30	52,70
Alcohol	0.25	0.96	0.36	1.27	0.64	------	90,20	9,80
Days without breakfast	0.71	1.62	0.74	1.68	0.09	22,90	77,10	------

Values are expressed as mean and standard deviation (SD) and adequacies are expressed in % according to SENC recommendations. Servings recommendation for males and females are the same in this age group.

**Fig 3:**
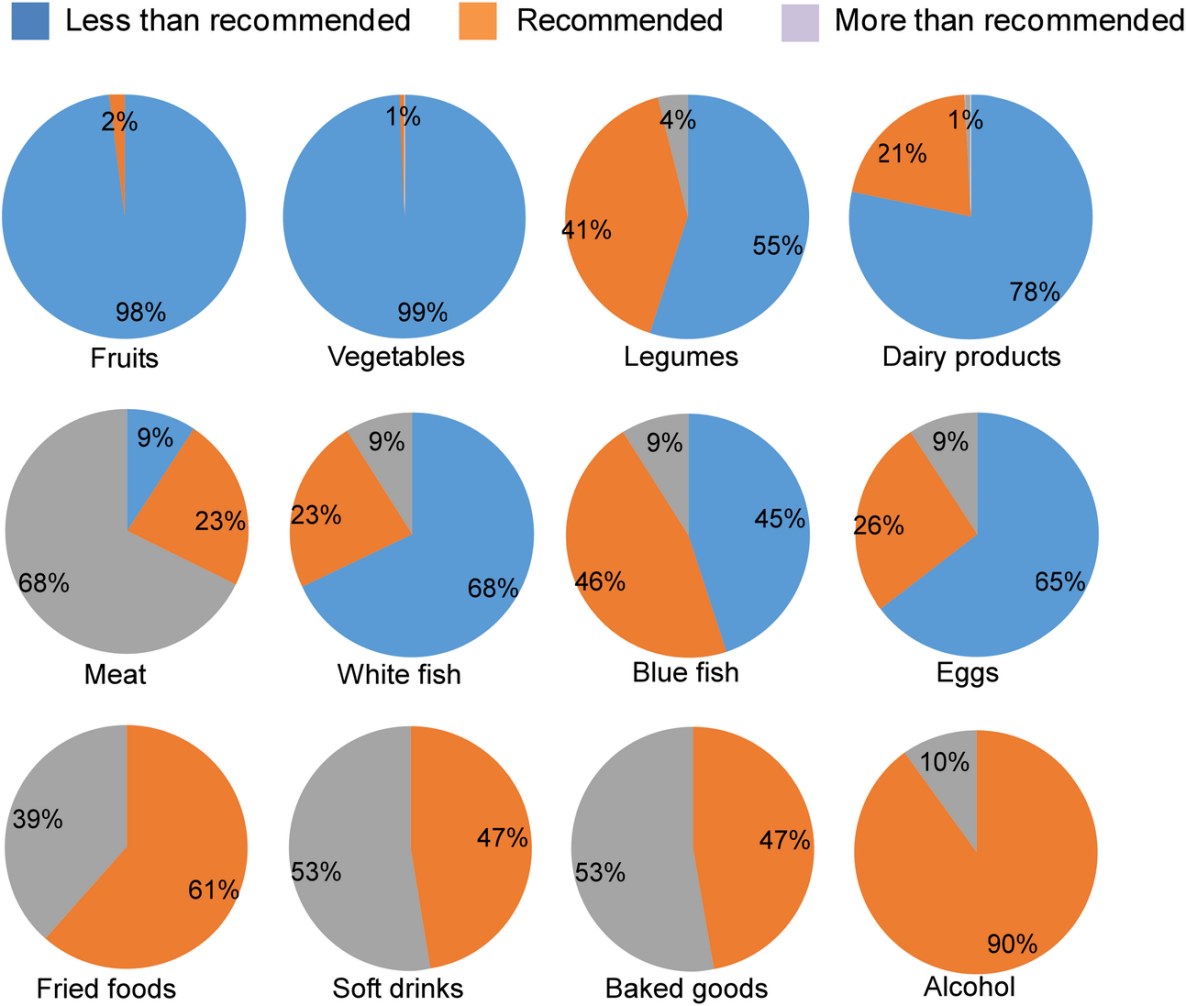
Compliance with dietary recommendations. Percentage of participants consuming insufficient, recommended or excessive weekly servings of the indicated foods and beverages. Reference values are provided by SENC dietary guidelines [32].

A group of nutritionists from Alicia Foundation examined the data and the uploaded photos of consumed meals to determine whether the number of servings corresponded to those recommended by the Spanish Society of Community Nutrition (SENC) [32]. **Fig 3** shows the percentage of students that met SENC recommendations. The majority of adolescents did not consume the minimal weekly recommended servings for fruits (98%), vegetables (99%), legumes (55%), dairy products (78%), white fish (68%) and eggs (65%). Moreover, about 40% or more of adolescents had a consumption greater than recommended for meat (68%), fried foods (39%), soft drinks (53%) and commercial baked goods (53%). Finally, a low intake of alcohol was observed, although approximately 10% of adolescents affirmed that they had consumed at least one alcoholic beverage during the week (data not shown). No statistically significant differences were observed between females and males regarding the respect of SENC recommendations, except for meat consumption, since more male adolescents ingested amount of meat higher than recommended compared to their female peers (70.3% vs 65.6%, p<0.05).

On the other hand, when adolescents were asked to specify how often they had breakfast over the week, 77% of them displayed good habits since they had breakfast every morning (data not shown).

The study of the macronutrients and micronutrients consumed by adolescents over one week (**Table 2**) highlights that females had a higher intake of proteins (%:17.40 ± 3.44 vs 13.82± 2.61, p = 0.0001) and carbohydrates (%:44.21 ± 6.45 vs 42.25± 4.96, p = 0.0001), whereas males consumed more total fatty acids (FAT) (%:43.75 ± 4.35 vs 38.13± 5.78, p = 0.001), saturated fatty acids (SFAT) (%:17.26 ± 2.17 vs 14.05± 2.77, p = 0.001), and polyunsaturated fatty acids (PUFA) (%:9.93 ± 1.89 vs 6.56± 2.21, p = 0.001),. Of note, Spanish adolescents, regardless their sex, ingested more proteins, FAT and MUFA on average than recommended. In addition, the consumption of sugars and SFAT was much higher than that indicated by SENC [32].

On the other hand, whereas the intake of micronutrients was comparable between genders, they both presented a low intake of vitamin D and calcium, considering SENC guidelines [32].

**Table 2:** Amount of macronutrients and micronutrients consumed over a week.

	Female		Males		
	Mean	SD	Mean	SD	p-value
Macronutrients*					
Proteins	17.40	3.44	13.82	2.61	0.0001
Carbohydrates	44.21	6.45	42.25	4.96	0.0001
Sugars	18.98	6.06	18.18	4.83	0.696
FAT	38.13	5.78	43.75	4.35	0.0001
SFAT	14.05	2.77	17.26	2.17	0.0001
MUFA	17.51	3.43	16.57	2.68	0.177
PUFA	6.56	2.21	9.93	1.89	0.0001
Micronutrients**					
Ca (mg)	728.60	249.07	613.88	281.01	0.65
Fe (mg)	12.62	6.64	10.70	6.52	0.68
Vitamin B6 (mg)	2.81	0.82	2.33	0.81	0.76
Vitamin D (mcg)	3.03	2.60	2.56	2.55	0.68
Vitamin B12 (mcg)	4.11	2.86	3.63	3.42	0.11
Vitamin C (mg)	84.53	51.46	73.97	54.19	0.27

Data are expressed as mean and standard deviation (SD).

*Values for macronutrient are expressed as percentages referring to total ingested Kcal.

** Values for micronutrients are adjusted to total ingested Kcal

### Association between BMI and PAQ-A score

To establish whether higher BMI values in overweight and obese adolescents were associated to reduced PA, the overall PAQ-A scores were evaluated in the four weigh categories.

**Fig 4:**
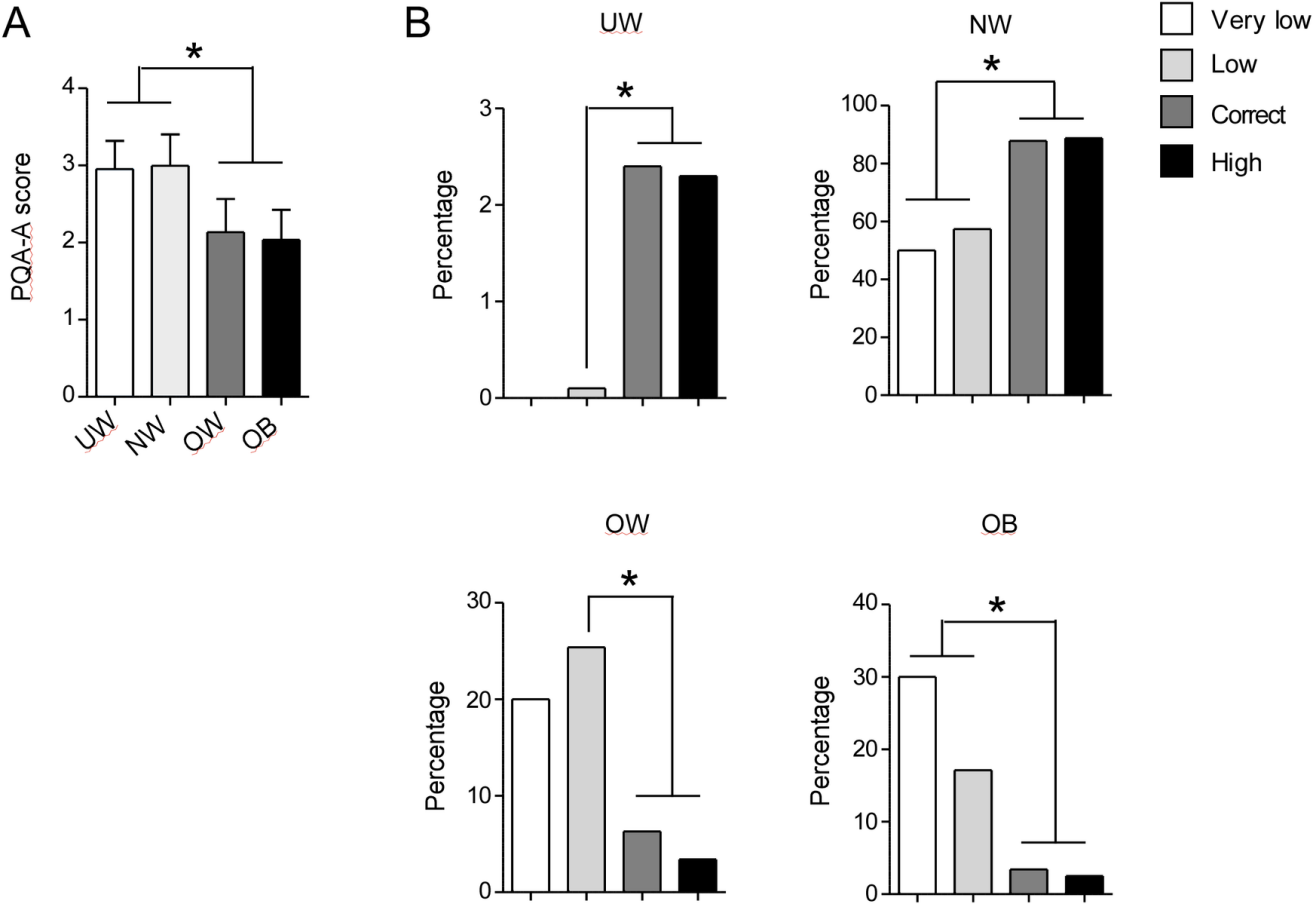
Association between BMI and PQA-A value. (A) PAQ-A scores obtained by participants grouped according to their BMI. Data are expressed as mean ± SD. (B) Percentage of UW (upper, left), NW (upper, right), OW (lower, left), OB (lower, right) participants grouped according to the their PAQ-A score. UW: underweight, NW: normal weight, OW: overweight, OB: obese. P<0.05

The analysis indicates that overweight and obese adolescents presented lower PAQ-A scores compared to underweight and normal weight participants (UW: 2.95 ± 0.64; NW: 2.99± 0.71; OW: 2.13± 0.74, OB: 2.03 ± 0.68, p<0.05), (**Fig 4A**).

Then, the percentage of underweight, normal weight, overweight and obese adolescents was analyzed in the four PAQ-A score categories corresponding to very low, low, correct and high PA.

Higher percentage of underweight subjects was found in adolescents practicing correct (2.4%) or high PA (2.3%) compared to low PA (0.1%). In addition, the percentage of normal weight adolescents was higher for PAQ-A scores ≥2 (correct and high PA, 87.9% and 88.8%, respectively, p<0.05) than for PAQ <2 (very low or low PA, 50% and 57.4%, respectively, p<0.05). Finally, the frequency of overweight and obese participants was statistically higher in very low (20% and 30%, respectively, p<0.05) and/or low PA (25.4% and 17.1%, respectively, p<0.05) groups compared to correct (6.3% and 3.4%, respectively, p<0.05) and high PA categories (3.4% and 2.5%, respectively, p<0.05) (**Fig 4B**).

### Association between weight and food, beverage and nutrient consumption

In order to identify an association between BMI and beverage, food and nutrient intake, the number of consumed servings and the amount of ingested calories and nutrients were analyzed in adolescents grouped according to their BMI.

**Fig 5:**
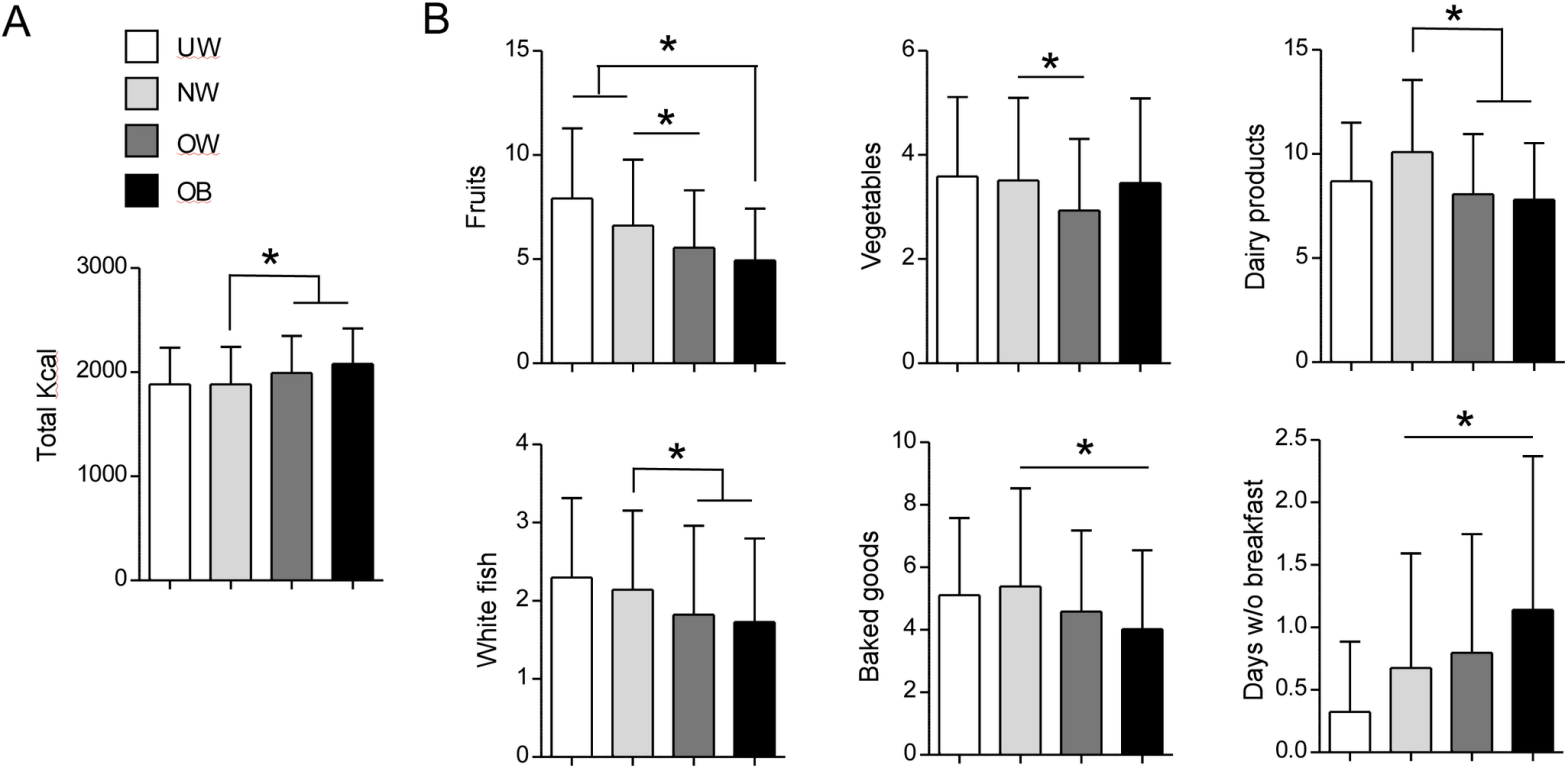
Association between BMI and food and beverage consumption. (A) Amounts of calories ingested by participants grouped according to their BMI. Data are expressed as mean ± SD. (B) Number of consumed weekly servings and number of days without breakfast in participants grouped according to their BMI. Data are expressed as mean ± SD. UW: underweight, NW: normal weight, OW: overweight, OB: obese. P<0.05

Data in **Fig 5A** and **Table 3** show that overweight and obese adolescents consumed on average more calories (1993 ± 613 and 2080 ± 590 Kcal/day, respectively) than their normal weight peers (1883 ± 622 Kcal/day, p<0.05). Moreover, overweight and/or obese subjects consumed less fruits (%: 5,56 ± 4,76 and 4,95± 4,28, respectively), vegetables (%: 2.93 ± 2.4 and 3.46 ± 2.8, respectively), dairy products (%: 8.07 ± 5 and 7.81 ± 4,7, respectively),and white fish (%: 1.82 ± 1.97 and 1.73± 1.85, respectively), compared to normal weight adolescents (%, fruits: 6.62 ± 5.48; vegetables: 3,51 ± 2.74;dairy products: 10,09 ± 6; white fish: 2.14 ± 1.76, p<0.05), but surprisingly less baked goods (%,OW:.4.58 ± 4.49; OB: 4.02 ± 4.38; NW: 5.39 ± 5.44, p<0.05). Finally, when the number of days without breakfast was evaluated, the results indicate that obese adolescents had breakfast less regularly than normal weight peers (1.14 ± 2.13 vs 0.68 ± 1.59, p<0.05) **(Fig 5B)**.

**Table 3:** Food and beverage intake in Spanish adolescents grouped according to their BMI and PAQ-A score.

	BMI			PA (PAQ-A score)		
	Category	Mean	SD	Category	Mean	SD
Fruits	UW	7.92^a^	5.83	Very low	3.50^d^	3.10
	NW	6.62^a^^,^^b^	5.48	Low	5.54^d^^,^^e^	4.82
	OW	5.56	4.76	Correct	6.65	5.34
	OB	4.95	4.3	High	7.60	6.39
Vegetables	UW	3.59	2.64	Very low	2.00^d^	1.56
	NW	3.51^b^	2.74	Low	3.18^d^^,^^e^	2.58
	OW	2.93	2.39	Correct	3.56	2.75
	OB	3.46	2.82	High	3.82	2.97
Legumes	UW	2.08	1.99	Very low	0.75^d^	0.79
	NW	1.60	1.57	Low	1.47^d^	1.63
	OW	1.49	1.38	Correct	1.6^d^	1.56
	OB	1.66	2.14	High	2.04	1.9
Dairy products	UW	8.70	4.86	Very low	5.80^e^	2.84
	NW	10.09^a^^,^^b^	6.0	Low	6.19^e^	3.4
	OW	8.07	4.99	Correct	6.86	3.79
	OB	7.81	4.70	High	6.50	3.676
Meat	UW	6.27	3.44	Very low	6.6	5.1
	NW	6.69	3.71	Low	8.7^e^	5.43
	OW	6.32	3.48	Correct	10.07	5.98
	OB	6.13	3.44	High	9.81	6.08
White fish	UW	2.30	1.76	Very low	0.80^d^^,^^e^^,^^f^	1.00
	NW	2.14^a^^,^^b^	1.76	Low	1.89^e^	1.88
	OW	1.82	1.98	Correct	2.18	1.78
	OB	1.73	1.85	High	2.12	1.67
Blue fish	UW	0.81	0.97	Very low	0.55	0.83
	NW	0.97	1.17	Low	0.83^d^	1.2
	OW	0.77	1.41	Correct	0.97	1.15
	OB	0.93	1.19	High	1.16	1.47
Eggs	UW	2.32	2.05	Very low	2.25	2.02
	NW	2.22	1.91	Low	2.11	1.84
	OW	2.11	2.1	Correct	2.23	1.919
	OB	2.26	2.19	High	2.36	2.389
Fried foods	UW	3.65	3.43	Very low	3.10	3.323
	NW	3.45	2.93	Low	3.33	2.695
	OW	3.51	2.96	Correct	3.46	3.041
	OB	3.11	2.55	High	3.47	2.836
Soft drinks	UW	4.65	5.11	Very low	4.50	3.532
	NW	5.24	5.33	Low	4.84	4.826
	OW	4.76	4.99	Correct	5.30	5.425
	OB	5.55	5.36	High	5.74	5.831
Baked goods	UW	5.11	4.28	Very low	6.40	6.116
	NW	5.39^a^	5.44	Low	5.01	4.818
	OW	4.58	4.5	Correct	5.18	5.256
	OB	4.02	4.38	High	5.36	6.092
Alcohol	UW	0.32	1.33	Very low	0.10	0.308
	NW	0.28	1.35	Low	0.25	0.913
	OW	0.27	0.99	Correct	0.29	1.418
	OB	0.58	2.83	High	0.51	2.687
Days without breakfast	UW	0.32	0.97	Very low	1.75^d^^,^^e^	2.573
	NW	0.68^a^	1.59	Low	0.86^e^	1.819
	OW	0.80	1.64	Correct	0.64	1.542
	OB	1.14	2.13	High	0.64	1.396

a: p<0.05 vs. OB adolescents

b: p<0.05 vs. OW adolescents.

d: p<0.05 vs. high PA

e: p<0.05 vs. correct PA

f: p<0.05 vs. low PA.

BMI: body mass index; PA: physical activity; UW: underweight; NW: normal weight; OW: overweight; OB: obese

The analysis of the intake of macronutrients indicates that obese and/or overweight adolescents ingested higher amounts of sugars (%:15.16 ± 4.62 and 15.37± 4.01, respectively), total FAT (%:41.40 ± 6.88 and 40.01 ± 6.58, respectively) and SFAT (%:21.17 ± 3.74 and 20.41 ± 3.52, respectively) than normal weight (% sugar: 12.32 ± 5.45; FAT: 38.87 ± 7.71; SFAT: 12.25 ± 3.66, p<0.05) and/or underweight peers (% sugar: 10.56 ± 6.38; FAT: 37.32 ± 7.32; SFAT: 12.28 ± 3.7, p<0.05). By contrast, obese and/or overweight participants had less proteins (%:8.46 ± 3.84 and 8.60 ± 3.44, respectively) carbohydrates (%:34.71 ± 4.32 and 35.76 ± 4.88, respectively), MUFA (%:11.55 ± 3.4 and 11.42 ± 3.71, respectively) and PUFA (%:8.70 ± 2.78 and 8.18 ± 2.44, respectively) than normal weigh adolescents (%, proteins: 9.37 ± 3.94; carbohydrates: 39.24 ± 5.72; MUFA: 12.44 ± 3.84; PUFA: 9.19 ± 3.10, p<0.05) (**Table 4**).

**Table 4:** Nutrient intake in Spanish adolescents grouped according to their BMI and PAQ-A score.

	BMI			PA (PAQ-A score)		
	Category	Mean	SD	Category	Mean	SD
Proteins	UW	10.07	3.95	Very low	8.49	3.59
	NW	9.37^a^^,^^b^	3.94	Low	8.93	3.82
	OW	8.60	3.44	Correct	9.33	3.93
	OB	8.46	3.84	High	9.44	3.77
Carbohydrates	UW	41.49^a^^,^^b^	6.13	Very low	36.75	4.50
	NW	39.24^a^^,^^b^	5.72	Low	37.85^d^^,^^e^	5.62
	OW	35.76	4.88	Correct	38.75	5.76
	OB	34.71	4.32	High	39.21	5.94
Sugars	UW	10.56^a^^,^^b^	6.38	Very low	13.63	5.76
	NW	12.32^a^^,^^b^	5.45	Low	13.60^d^^,^^e^	5.09
	OW	15.37	4.01	Correct	12.59	5.53
	OB	15.16	4.62	High	12.32	5.25
FAT	UW	37.32^a^	7.32	Very low	41.14	7.78
	NW	38.87^a^	7,71	Low	39.39	7.11
	OW	40.01	6.58	Correct	39.10	7.66
	OB	41.40	6.82	High	38.87	7.99
SFAT	UW	12.28^a^^,^^b^^,^^c^	3.70	Very low	18.92	3.84
	NW	17.25^a^^,^^b^	3.66	Low	18.48^d^^,^^e^	4.03
	OW	20.41	3.52	Correct	17.54	3.84
	OB	21.17	3.74	High	17.46	4.17
MUFA	UW	14.23^a^^,^^b^^,^^c^	4.06	Very low	12.56	3.25
	NW	12.44^a^^,^^b^	3.84	Low	12.03	3.62
	OW	11.42	3.71	Correct	12.40	3.88
	OB	11.55	3.40	High	12.46	4.17
PUFA	UW	10.83^a^^,^^b^^,^^c^	3.85	Very low	9.66	3.64
	NW	9.19^b^	3.10	Low	8.88	2.86
	OW	8.18	2.44	Correct	9.16	3.13
	OB	8.70	2.78	High	8.95	2.94

a: p<0.05 vs. OB adolescents

b: p<0.05 vs. OW adolescents

c: p<0.05 vs. NW adolescents.

d: p<0.05 vs. high PA

e: p<0.05 vs. correct PA.

BMI: body mass index; PA: physical activity; UW: underweight; NW: normal weight; OW: overweight; OB: obese.

Analysis performed on females and males for separately did not show statistically significant differences in the consumption of the majority of macronutrients among BMI or PAQ-A categories, However, underweight females and/ or females with a normal weight consumed less proteins (%: 8.86 ± 3.10 and 8.15± 3.18, respectively) and carbohydrates (40.77 ± 5.52 and 37.88 ± 4.34, respectively) than overweight (%, proteins: 7.75 ± 2.79; carbohydrates: 35.33 ± 3.87, p<0.05) and/or obese (%, proteins: 7.11 ± 3.10; carbohydrates: 34.72 ± 3.7, p<0.05) peers (**Table 5** and **Table 6**).

**Table 5:** Nutrient intake in BMI and PAQ-A categories in females.

	BMI			PAQ-A		
	Category	Mean	SD	Category	Mean	SD
Proteins	UW	8.86	3.1	Very low	7.02	3.07
	NW	8.15^a^	3.18	Low	7.76	3.05
	OW	7.75	2.79	Correct	8.14	3.16
	OB	7.11	3.1	High	8.26	3.39
Carbohydrates	UW	40.77^a^^,^^b^^,^^c^	5.52	Very low	35.77	4.79
	NW	37.88^a^^,^^b^	4.34	Low	37.06	4.21
	OW	35.33	3.87	Correct	37.59	4.47
	OB	34.72	3.75	High	37.64	4.89
Sugar	UW	11.15	5.24	Very low	14.34	6.24
	NW	12.43	4.75	Low	13.73	4.33
	OW	14.89	3.95	Correct	12.55	4.91
	OB	15.42	3.62	High	12.43	4.28
FAT	UW	38.62	6.61	Very low	42.87	8.1
	NW	41.37	6.48	Low	41.27	5.93
	OW	41.93	5.53	Correct	41.54	6.43
	OB	42.53	5.24	High	41.58	6.37
SFAT	UW	13.55	2.45	Very low	19.17	4.52
	NW	18.4	3.21	Low	19.27	3.39
	OW	20.92	3.1	Correct	18.64	3.37
	OB	21.31	3.04	High	18.53	3.53
MUFA	UW	13.48	3.46	Very low	13.19	3.1
	NW	12.37	3.24	Low	11.85	3.05
	OW	11.45	3.03	Correct	12.27	3.28
	OB	11.13	2.88	High	12.72	3.37
PUFA	UW	11.58	3.4	Very low	10.52	3.77
	NW	10.59	2.62	Low	10.15	2.39
	OW	9.56	1.87	Correct	10.63	2.61
	OB	10.08	2.19	High	10.34	2.38

a: p<0.05 vs. OB adolescents

b: p<0.05 vs. OW adolescents

c: p<0.05 vs. NW adolescents.

BMI: body mass index; PA: physical activity; UW: underweight; NW: normal weight; OW: overweight; OB: obese.

**Table 6:** Nutrient intake in BMI and PAQ-A categories in males.

	BMI			PAQ-A		
	Category	Mean	SD	Category	Mean	SD
Proteins	UW	12.3	4.48	Very low	10.69	3.3
	NW	10.92	4.25	Low	10.42	4.17
	OW	9.57	3.84	Correct	10.85	4.29
	OB	10.39	3.99	High	10.89	3.72
Carbohydrates	UW	42.81	7.18	Very low	38.22	3.87
	NW	40.96	6.72	Low	38.87	6.89
	OW	36.25	5.8	Correct	40.23	6.8
	OB	34.68	5.06	High	41.13	6.54
Sugar	UW	9.45	8.21	Very low	12.55	5.17
	NW	12.17	6.22	Low	13.43	5.92
	OW	15.93	4.02	Correct	12.64	6.23
	OB	14.78	5.77	High	12.19	6.26
FAT	UW	34.95	8.23	Very low	38.54	6.97
	NW	35.69	7.97	Low	36.98	7.75
	OW	37.83	7.03	Correct	35.97	7.98
	OB	39.81	8.38	High	35.57	8.52
SFAT	UW	9.92	4.51	Very low	18.54	2.79
	NW	15.77	3.66	Low	17.47	4.52
	OW	19.84	3.87	Correct	16.14	3.95
	OB	20.96	4.57	High	16.16	4.52
MUFA	UW	15.6	4.84	Very low	11.62	3.45
	NW	12.52	4.49	Low	12.25	4.24
	OW	11.39	4.38	Correct	12.57	4.53
	OB	12.13	3.97	High	12.15	4.97
PUFA	UW	9.43	4.37	Very low	8.37	3.24
	NW	7.41	2.73	Low	7.25	2.58
	OW	6.6	2.02	Correct	7.26	2.7
	OB	6.71	2.29	High	7.25	2.67

BMI: body mass index; PA: physical activity; UW: underweight; NW: normal weight; OW: overweight; OB: obese.

### Association between physical activity and food, beverage and nutrient consumption

To assess whether insufficient PA was also accompanied by incorrect eating habits, the assumption of food and beverage, as well as the intake of macronutrients were compared in adolescents used to have very low, low, correct or high PA. Data indicate that the overall amount of ingested calories was comparable in very low, low, correct and PA groups (2182±652, 1963±631, 1889±613, and 1866±614 Kcal/day, respectively).

**Fig 6:**
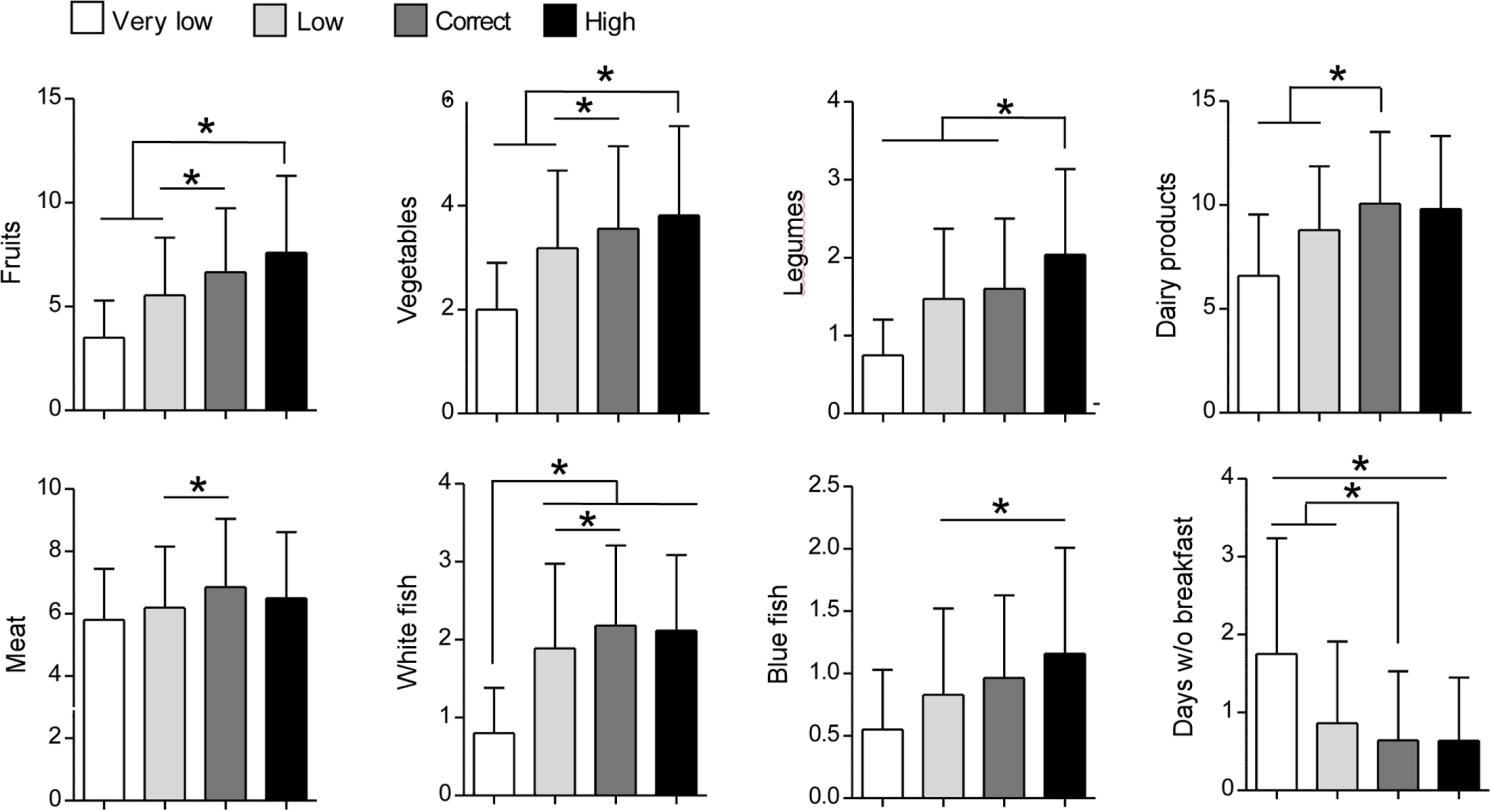
Association between PAQ-A score and food and beverage consumption. Number of consumed weekly servings and number of days without breakfast in participants grouped according to their PQA-A score. Data are expressed as mean ± SD.

**Fig 6** and **Table 3** indicate that adolescents engaged in very low or low PA also ingested less fruits (%: 3.50 ± 3.10 and 5.54 ±4.82, respectively) vegetables (%: 2 ± 1.56 and 3.18 ± 2.58, respectively), legumes (%: 0.75 ± 0.79 and 1.47 ± 1.63, respectively), dairy products (%: 5.80 ± 2.84 and 6.19 ± 3.40, respectively), meat (%: 6.6 ± 5.1 and 8.7 ± 5.43, respectively), white fish (%: 0.80 ± 1.0 and 1.89 ± 1.88, respectively), blue fish (%: 0.55 ± 0.83 and 0.83 ± 1.12, respectively) than adolescents with a correct (%, fruits: 6.65 ± 5.34; vegetables: 3.56 ± 2.75; legumes: 1.6 ± 1.56; dairy products: 6.86 ± 3.79; meat: 10.07 ± 5.97; white fish: 2.18 ± 1.67; blue fish: 0.97 ± 1.15, p< 0.05) or high PA (%, fruits: 7.60 ± 6.39; vegetables: 3.83 ± 2.97; legumes: 2.04 ± 1.9; dairy products: 6.5 ± 3.68; meat: 9.81 ± 6.08; white fish: 2.12 ± 1.67; blue fish: 1.16 ± 1.47, p< 0.05) In addition, they also had breakfast less regularly (number of days w/o breakfast: very low PA: 1.75 ± 2.57; low PA: 0.86 ± 1.82; correct PA: 0.64 ± 1.54; high PA: 0.64 ± 1.4) The consumption of eggs, fried foods, soft drinks and commercial baked goods was comparable in all the groups.

Furthermore, participants practicing low PA also showed reduced intake of carbohydrates (%:37.85 ± 5.62) but higher assumption of sugars (%:13.60 ± 5.09) and SFAT (%:18.48 ± 4.03) than their peers engaged in correct (%, carbohydrates: 38.75 ± 5.76; sugars: 12.59 ± 5.53; SFAT: 17.54 ± 3.84, p<0.05) or high PA activity (%, carbohydrates: 39.21 ± 5.94; sugars: 12.32 ± 5.25; SFAT: 17.46 ± 4.17, p<0.05) (**Table 4**).

## Discussion

The purpose of this cross-sectional study was to give an updated picture of the eating habits and the PA adopted by Spanish adolescents. Because of the paucity of educational programmes aimed to investigate and improve healthy habits in adolescents in Spain, the CAL-TAS program was launched by Alicia Foundation in 2011, in order to identify interventional strategies against obesity. The main strength of this study is the choice of an age range (13–14 years) that is critical to determine the state of health in the adult age. During these years, adolescents acquire more independence and make their own food choices [20]. In addition, they are still under the umbrella of compulsory education, which permits the elaboration of targeted interventional strategies aimed to established healthy diet and proper PA that are critical to ensure a correct growth and development.

We observed that many of the children who just entered into adolescence already had incorrect eating habits. We indeed observed that 20% of the participants were overweight or obese. In addition, the analysis of the diet adopted by Spanish adolescents indicated that a considerable fraction of them did not respect SENC recommendations. A high consumption of meat, fried foods and commercial baked goods was observed in the considered population. By contrast, the intake of fruits, vegetables, and white fish was lower than that recommended. As expected, overweight and/or obese adolescents presented pretty unhealthy dietary habits and practiced reduced PA compared to subjects with a normal weight.

Similar previous studies aimed to determine the prevalence of overweight and obese adolescents in Spain achieved comparable results. The enKid study evaluated the food habits of Spanish children and youths aged 2 to 24 years. The authors concluded that 26.3% of the sample population was overweight or obese, that the prevalence of obesity was more common in male, in children aged 6–13 years, in people with lower socioeconomic or educational level and that obesity was often associated with no or poor breakfast consumption [34].

Similarly, the AVENA study was designed to investigate the prevalence of overweight and obese adolescents from five Spanish cities. The collected data indicated an elevated overweight and obese prevalence in Spanish adolescents, similar to those observed in other European countries. Interestingly, overweight and obese prevalence is increasing in the last years and in this settings seems to be related to a socioeconomic status in males, but not in females [35].

Obesity in children and adolescents has a multifactorial nature, although it can be principally ascribed to unhealthy dietary habits, lack of PA and sedentary behavior.

Regular PA is important for young people’s health, since it is associated with a healthier and longer life [36,37], protects from cardiovascular disorders [38] and has a positively influence on weight regulation [39,40]. We found that overweight and obese adolescents presented lower PAQ-A scores compared to underweight and normal weight participants and that the percentage of overweight and obese subjects was higher in groups practicing low or very low PA. Although adolescents are known to be less active than children [41], their PA is generally more structured. On the other hand, a systematic review of the existing literature highlights that PA declines during adolescence, with females and males presenting different trends [42]. In particular females start to reduce their PA earlier compared to males, and PA decrease is associated with higher BMI [43].

In this regard, our data are in line with previous results obtained by Aznar et al. from a sample of Spanish 15-year old adolescents, whose PA was measured using the GT1M accelerometer [41]. Accordingly, we show that females presented lower PAQ-A values and were generally less active than males. This difference could be ascribed to the fact that females are generally less engaged in extra scholar sport activities than their male peers. For this reason, adolescents would benefit from effective intervention incorporating PA not only at school but also after school and over the weekend.

We observed that adolescents engaged in very low or low PA also presented less healthy dietary habits, suggesting that different diet and activity variables, together with diverse socioeconomic conditions, may interplay and determine the overweight status. In this respect, most of the studies only consider the association between either food ingestion or PA or sedentary behavior. The CAL-TAS program, by contrast, is a multivariate project that investigates distinct eating and PA behavior patterns. A similar approach was adopted by Patrick et al. who examined the relationship between BMI, diet and PA in a group of adolescents from six clinic sites in San Diego Country. The authors concluded that inadequate vigorous PA was the only risk factor for higher BMI in adolescents. However, they did not exclude that the use of self-report for dietary behavior may have affect their conclusions [40].

Our observation that Spanish adolescents scarcely follow guidelines for diet is supported by previously published data. HELENA is a study encompassing more than three thousand adolescents aged 12.5–17.5 years from all over Europe. This study disclosures a critical imbalance in the intake of foods and nutrients, since the consumption of the recommended amount of fruits, vegetables and dairy products was shown to be almost halved. By contrast, the intake of meat products, fats, and sweets was higher than recommended [44,45]. Adolescents are highly vulnerable to the adoption of unhealthy dietary habits, as extensively showed by the study ANDALIES, performed with students from secondary school of Andalusia. Indeed, the elevated intake of sugars and fats is encouraged by the lack of activities for the promotion of healthy eating habits and the low quality of the food offered by the cafeterias [46]. The adherence to Mediterranean diet, considered a model of healthy diet, decreased with age: younger adolescents display higher consumer of fruits, vegetables and fish compared to older adolescent subjects [47].

We also found that Spanish adolescents did not properly follow recommendations for macronutrient and micronutrient intake. In particular, obese and overweight participants, as well as subjects practicing low PA, ingested more sugars and SFAT, but less proteins and complex carbohydrates than adolescents with normal weight or engaged in normal PA. Of note, we also observed that although the total amount of ingested calories was correct, adolescents tended to consume more proteins, sugars, and fats (especially MUFA and SFAT), but less vitamin D and calcium than they should. A recent dietary survey (ANIBES study) performed with a nationally representative sample of Spanish population (aged 9–75 years) claims that adolescents have the highest energy intake, mainly provided by carbohydrates, fats and proteins. Furthermore, similarly to what we observed from our cohort of subjects, males present a mean energy intake higher than females. The same study also shows a decrease in energy intake in the overall Spanish population and a progressive distancing from the Mediterranean diet [48].

The HELENA study provides comparable results for European adolescents, who intake high quantity of SFAT and salt and few amounts of PUFA. In the same sample, also an intake of vitamin D < 50% of reference value is observed [44,45].

In our study, most of the participants had breakfast regularly. However, overweight and obese subjects were more prone to skip it. Breakfast provides 25% of the daily energy intake and several publications propose that a regular breakfast consumption is associated with a reduced risk of obesity, a lower BMI and high academic achievement [49–51]. Though, breakfast-eating frequency is reported to decline through adolescence.

A study carried out with Spanish adolescents from 1^st^ grade shows that students having an insufficient quality breakfast also have lower school performance [52]. In addition, irregular breakfast consumption is associated with low frequency of fruit intake in adolescence, and diets high in fruits and vegetables are known to reduce the risk of cardiovascular diseases [53,54].

In Canada, adolescents from 59 public and private schools participated to a web-based survey in order to study their diet and PA. The participants were classified as overweight, obese and non-overweight. Analogously to our findings, overweight and obese subjects, who corresponded to 21.1% of the students, have more fat intake than non- overweight peers and consume breakfast less regularly [55]. Our data and data from other groups thus support the importance of encouraging regular breakfast consumption among adolescents.

About half of adolescents participating to the CAL-TAS survey indicated that they consumed around five soft drinks over one week. The impact of sugared soft drink consumption during childhood and adolescence on the development of obesity and metabolic disorders, such as type 2 diabetes, has been debated worldwide in recent years [56,57]. In United States, sugar-sweetened beverages (SSB) represent the main source of energy intake, and are associated with higher waist circumference and low levels of HDL in blood [58]. In Europe, SSB are the second most commonly consumed beverage, after water. In Spain, sweetened milk drinks, low-fat milk and fruit juice are the beverages the most consumed by adolescents [59], and sugared soft drinks represent 3.4% of the total energy intake in adolescents, a higher ingestion compared to children and adults [48]. Even thought we could not detect a statistically significant difference in the intake of sugared soft drink between the adolescents grouped on the base of their BMI or PAQ-A score, we observed that overweight and obese adolescents, as well as participants with low PA, ingested more sugars than their peers with a normal weight or with a correct PA.

Although reducing SSB consumption alone would not probably solve the risk to develop obesity, limiting their intake may have a significant impact on the prevention of obesity-related disorders.

Several interventions have already been conducted throughout Europe and United States to promote PA between adolescents, in both scholar and family settings [60]. Unfortunately, the results obtained are quite heterogeneous in terms of sample size, duration, and measuring of overall activity. Moreover, they cannot be easily generalized from one country to another one. However, even though most of the interventions generally lack long-term follow-up, they seem to be more effective when they involve school, families and community [61]. In Spain, only thirteen school-based interventions have been proposed in the last fifteen years, in order to enhance PA or good dietary habits in adolescents. These programmes present the same limitations, even though they all led to an improvement of healthy habits [62].

The heterogeneity of the information provided by the different studies highlights the need of identifying the most effective strategies in order to draw more successful school- and family-based interventional programs.

Finally, the embracing of regular and healthy dietary habits, as well as the practice of PA during adolescence are associated not only with a reduction in obesity, but also with improved academic accomplishment [51,63].

The CAL-TAS program was designed to develop interventional strategies on the basis of the results obtained from the records of diet and PA, as well as on academic curriculum and adolescent age preferences. The final goal of this project was to improve both eating habits and PA through the passion for cooking and leisure activities.

The proposed study presents some limitations. Data obtained about dietary habits and PA engagement were self-reported by participants. Thus, we cannot exclude that some information was modified or omitted. However, we believe that the anonymous nature of the records minimized the risk of bias and encouraged honesty in the responses. On the other hand, since it is a cross-sectional study, we could not establish causality between the observed parameters. Therefore, it is necessary to carry out controlled experimental studies to confirm the observed associations. In addition, the socioeconomic status of the schools (and hence of the families) has not been considered in the analysis. In this regard, although we recluted adolescents distributed throughout the national territory, we cannot exclude that results may be influenced by the provenance of the students. The evaluation of this variables may be important and should be taken into consideration when designing future interventional progammes. Despite these limitations, the study was performed with a large and representative sample of the Spanish adolescent population, which allowed us to obtain a current and valid picture of consumer habits, nutritional status and PA of this population.

## Conclusions

Our analysis discloses a still too high percentage of overweight or obese adolescents in Spain and an overall non-compliance with SENC recommendation. Moreover, the participants with higher BMI were also the ones with the worst dietary habits and the highest physical inactivity. Transition from childhood to adolescence is usually characterized by a critical decline of healthy diet behavior, and can be predictive of future pathological conditions, but is also the period where young people need to become aware about their responsibility in their own food choice and the establishment of healthy habits. For this reason, knowledge and self-consciousness are key values that should be considered at the time of elaborating effective interventional programmes and that should be transmitted to adolescents.
